# Interdisciplinary dental management of patient with oligodontia and maxillary hypoplasia: a case report

**DOI:** 10.1186/s12903-022-02117-1

**Published:** 2022-03-22

**Authors:** Sharon Aronovich, Yuan-Lynn Hsieh, Richard Scott Conley, Bradley Stieper, Marilia Yatabe, Fei Liu

**Affiliations:** 1 Department of Oral and Maxillofacial Surgery, University of Michigan School of Dentistry, Ann Arbor, MI 48109 USA; 2 Department of Biologic and Materials Sciences & Prosthodontics, University of Michigan School of Dentistry, 1011 N University Ave., Ann Arbor, MI 48109 USA; 3 Department of Orthodontics and Pediatric Dentistry, University of Michigan School of Dentistry, Ann Arbor, MI 48109 USA; 4 Present Address: Division of Restorative and Prosthetic Dentistry, The Ohio State University, Columbus, OH 43210 USA; 5 Present Address: Department of Orthodontics, University of Missouri at Kansas City, Kansas City, MO 64108 USA; 6 Present Address: Stieper and Brust Orthodontics, 10460 Pelham Rd, Taylor, MI 48180 USA

**Keywords:** Interdisciplinary, Le Fort I maxillary osteotomy, Orthodontic, Dental implant, Prosthodontic, Case report

## Abstract

**Background:**

The craniofacial developmental abnormality can significantly complicate the oral rehabilitation of patients with oligodontia. This case report describes an interdisciplinary approach that took 7 years to successfully treat a young patient with non-syndromic oligodontia and midface deficiency.

**Case presentation:**

A 14-year-old patient with complex oral and maxillofacial conditions and diagnosis of oligodontia presented to our clinic. In addition to 4 retained deciduous teeth and congenitally missing 10 permanent teeth, dentofacial findings included maxillary and malar deficiency with a concave facial profile, Angle Class III malocclusion, and poor dental esthetics. The interdisciplinary treatment included pre-surgical orthodontic decompensation, high Le Fort I maxillary osteotomy, postsurgical orthodontic therapy, osseous ridge augmentation using recombinant human bone morphogenetic protein-2 (rhBMP-2), interim removable partial denture, dental implant installation, interim implant prostheses, and final prosthetic rehabilitation.

**Conclusions:**

The successful treatment of patients with oligodontia and complex dentofacial abnormalities requires the close and orderly collaboration among orthodontist, oral maxillofacial surgeon, and prosthodontist. Within the limitations of this case report, presented interdisciplinary approaches may optimize the oral rehabilitation outcome in patients with similar clinical challenges. A prospective clinical investigation is desired to verify the benefit of presented interdisciplinary approach.

## Background

Oligodontia refers to congenitally missing six or more teeth, excluding the third molars [1]. The prevalence of oligodontia is reported 0.14–0.3% of the general population [1, 2]. Although oligodontia often presents as an isolated trait, called non-syndromic oligodontia, it can also be part of a syndrome, called syndromic oligodontia. Ectodermal dysplasia is the most common group of syndromes that are associated with oligodontia. A diagnosis of ectodermal dysplasia may be clinically suspected based on abnormalities and symptoms related to ectoderm-derived structures. Skin, hair, sweat gland and teeth are the most vulnerable tissues in the affected subjects. Typical clinical manifestations are sparse hair, dry skin, lack of sweat gland, saddle nose, midface depression and absent of teeth [3, 4]. Gene mutations, drug-induced disturbance in tooth germ, and nutrition imbalances are common factors that are related to non-syndromic oligodontia [5, 6]. Paired box 9, ectodysplasin A, msh homeobox 1, axis inhibition protein 2, EDAR-associated death domain, NF-kappa-B essential modulator, and Keratin 17 are among the known genes whose mutations may potentially cause non-syndromic oligodontia [6].

Due to the adverse consequences on appearance and pronunciation, patients with oligodontia often suffer from psychosocial impact in their early childhood and adolescence, and thus, their oral health-related quality of life is significantly affected [7]. The oral rehabilitation of patients with oligodontia is often very complex because oligodontia is commonly associated with other changes in the orofacial complex, such as the morphology and size of the teeth, malocclusion, growth disturbances of the maxillofacial skeleton and thus the facial appearance, and insufficient bone for implant treatment [8–11].

Dental treatment of patients with oligodontia requires several special considerations. Firstly, since the dental defects are congenital, early dental intervention would greatly improve oral function and increase the health-related quality of life [12]. Moreover, studies have suggested the importance of implementing comprehensive dental treatment to satisfy the needs of children in tandem with their stage of growth and development [13, 14]. Comprehensive oral care for those afflicted with oligodontia typically includes early diagnosis, ideally at the preschool age, long-term planning, and interdisciplinary collaboration between various dental and medical providers. Specialties involved may include pediatric dentists, orthodontists, oral surgeons, periodontists and prosthodontists [13, 15, 16]. For restorative strategies, removable prosthesis is usually the first prescribed prosthesis to improve oral function in patients with oligodontia because it could be easily adjusted and rapidly refabricated to accommodate the change of the oral environment with continuous growth of jawbone and remaining teeth in young oligodontia patients [12, 13]. As the growth slows or ceases, pre-prosthetic preparation such as orthodontic therapy, orthognathic surgery and osseous ridge augmentation surgery are commonly considered in order to achieve optimal function and esthetics for the permanent prostheses [17–19]. Although dental treatments in this population are complex and challenging, under the comprehensive care provided by an experienced interdisciplinary dental team, patients may achieve satisfactory appearance, oral function and improved oral health-related quality of life [12].

In this case report, we present the successful oral rehabilitation of a female adolescent with oligodontia using dental implants. Before the implant surgery and final prosthetic restorations, the staged orthodontic therapy, orthognathic surgery, BMP2-assisted bone augmentation, provisional removable partial denture, provisional implant prostheses had been provided to optimize the clinical result.

## Case presentation

**Fig. 1:**
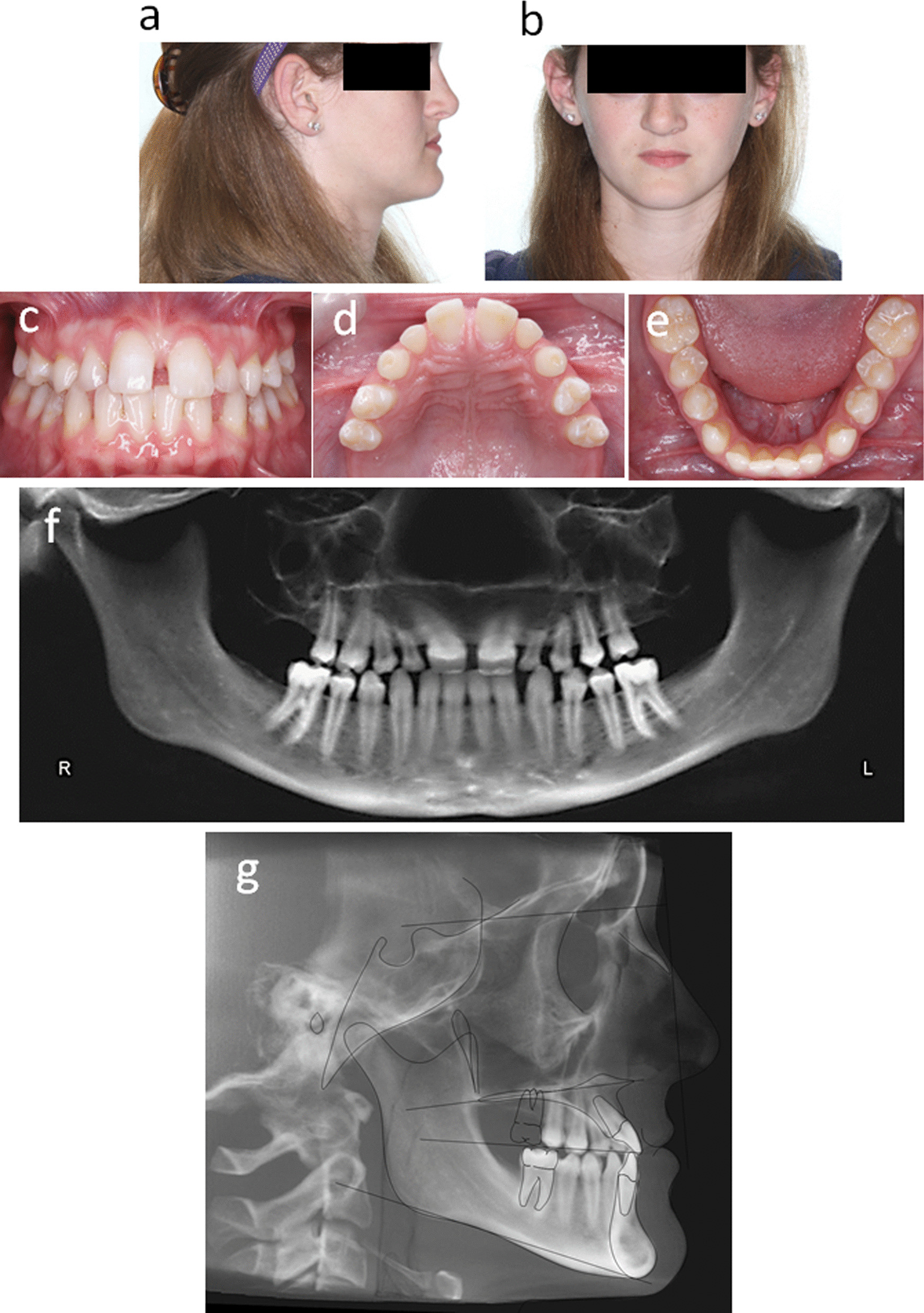
Initial examination before the treatment: **a** and **b** Extraoral photographs. **c**–**e** Intraoral photographs. **f** Panoramic radiograph. **g** The lateral cephalometric radiograph and tracing

The patient initially presented to the graduate orthodontic program at the University of Michigan School of Dentistry at 14 years of age with complaints of poor dental esthetics with diastema and abnormalities in the shape/size and alignment of front teeth, speech issues, and multiple missing teeth. Diagnosis of oligodontia was reported in one or more of her siblings. On examination, maxillary and malar hypoplasia was noted, along with a concave facial profile, maxillary asymmetry, a skeletal Class III malocclusion, and oligodontia (Fig. 1a–e). A panoramic radiograph confirmed the absence of teeth #1, 2, 3, 6, 7, 10, 11, 14, 15, 16, 17, 18, 31, 32, and the presence of retained primary teeth D, E, G, H (Fig. 1f). The lateral cephalometric radiograph and tracing revealed the underlying skeletal Class III nature of her malocclusion. (Fig. 1g). Since no abnormalities in other parts of the body were seen on exam, including nail, skin, hair, salivary and sweat glands, the patient was diagnosed with non-syndromic oligodontia with maxillary hypoplasia and malar deficiency. The patient was referred to the department of oral and maxillofacial surgery at the same institute for further evaluation. It was determined that orthognathic surgery was necessary to treat her maxillary hypoplasia and malar deficiency.

**Fig. 2:**
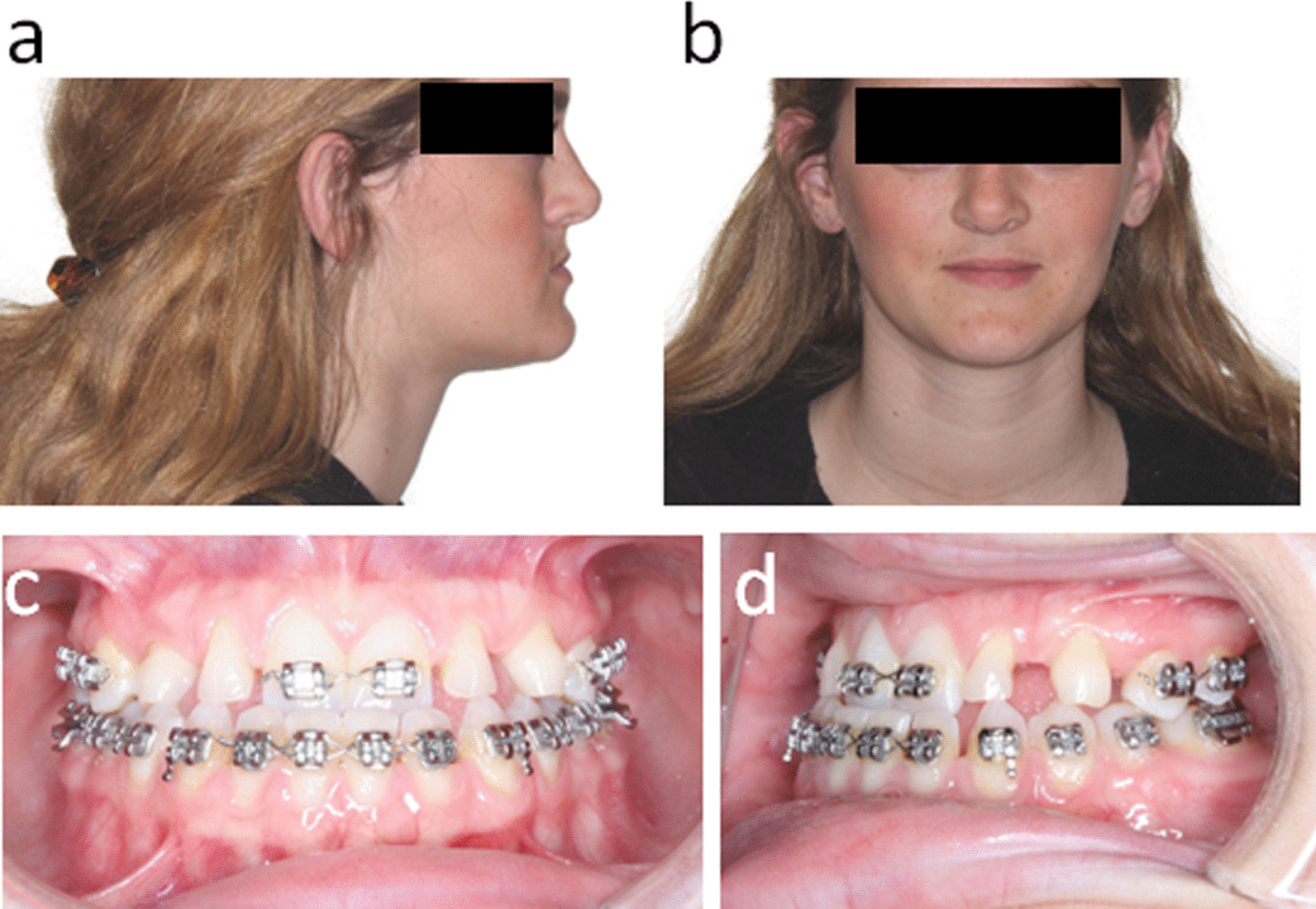
After the initial orthodontic treatment: **a** and **b** extraoral photographs. **c**, **d** intraoral photographs

**Fig. 3:**
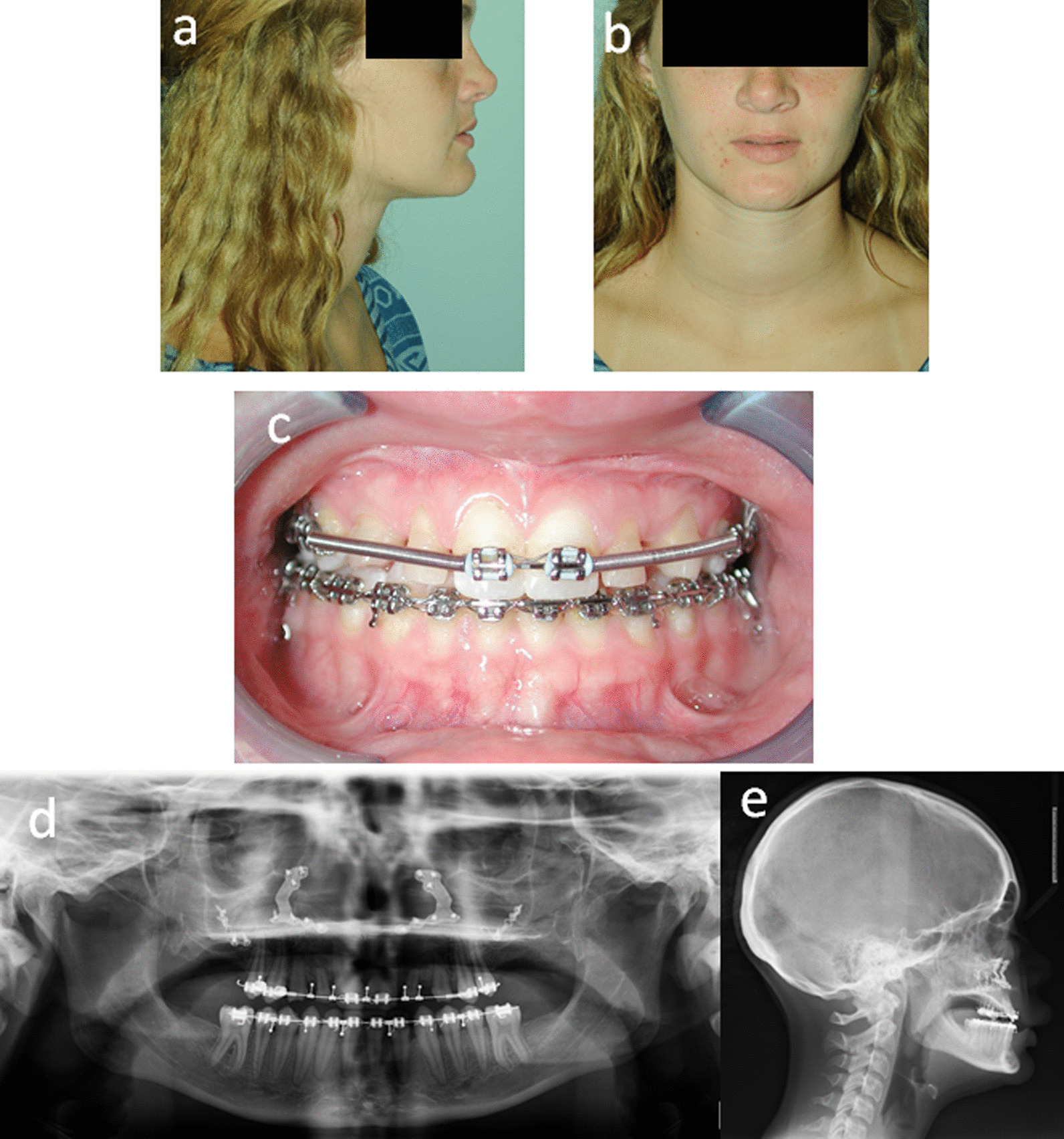
After the high Le Fort 1 maxillary orthognathic surgery: **a** and **b** Extraoral photograph. **c** Intraoral photograph. **d** Panoramic radiograph. **e** Cephalomatric radiographs

As a result of the compensatory response to the skeletal Class III condition, the patient had flared maxillary incisors with diastema (Fig. 1c, d). Thus, pre-surgical orthodontic treatment was carefully planned with multidisciplinary inputs to facilitate future orthognathic surgery and prosthodontic needs (Fig. 2). The goal of this phase of treatment was to retract and upright the patient’s anterior teeth into the ideal position in the alveolar base, and allow a proper occlusion after the jaws are aligned. As expected, the pre-surgical orthodontic decompensation treatment closed the diastema and led to a slight reverse overjet with coincident dental to facial midlines (Fig. 2c, d). Next, at the age of 17, a high Le Fort I maxillary osteotomy was performed to advance the maxilla. This operation successfully improved the midface projection (Fig. 3a, b), achieved positive overjet and overbite (Fig. 3c). Post-operative radiographs demonstrate the new maxillary position with fixation at the pyriform rims and zygomatic buttresses (Fig. 3d, e).

**Fig. 4:**
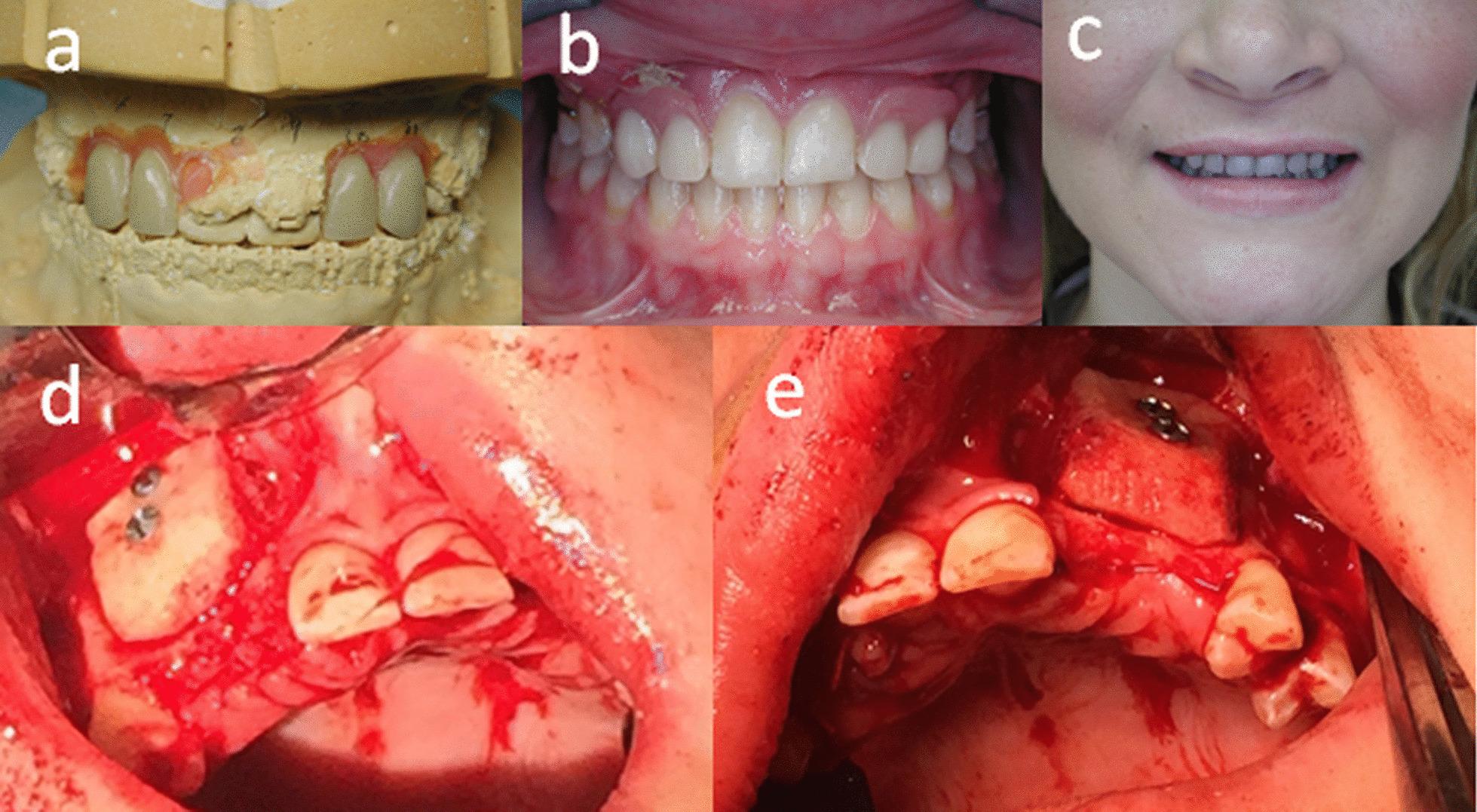
Preparation for implant surgery: **a** Diagnostic wax-up in # 6, 7, 10 and 11 for initial prosthodontic planning and space analysis. **b** and **c** Interim partial denture insertion after the extraction of retained primary teeth. **d** and **e** Allograft block bone grafts were fixed on the labial side of #6, 7, 10 and 11

After the orthognathic surgery, orthodontic treatment was continued to refine the alignment, root parallelism, and coordinate the arches with guidance from the prosthodontist. One goal of post-operative orthodontic treatment was to provide the optimized restorative space for implant prostheses. Diagnostic teeth set-up was performed to confirm the proper space and project future teeth position (Fig. 4a). The planned tooth size and shape were communicated with patient to her satisfaction. Thereafter, retained primary teeth C, D, G, and H were extracted and a particulate allograft was used for ridge preservation with a cross-linked collagen membrane. Interim maxillary partial denture was fabricated for the esthetics and maintenance of space (Fig. 4b, c). A few months later, the patient presented with inadequate ridge width in the edentulous maxillary ridge areas. Additionally, the alveolar ridge surrounding adjacent permanent teeth was also very thin. The maxillary rigid fixation was noted to pose a potential interference to future implant placement and apical extension of bone graft material. Therefore, the maxillary anterior rigid fixation was removed and an allograft block grafting was performed at sites #6, 7 and 10, 11 and secured with lag screws (Fig. 4d, e). The particulate allograft with the use of recombinant human bone morphogenetic protein-2 (rhBMP-2) (Infuse™ bone graft) was applied peripherally on either side of the block graft.

**Fig. 5:**
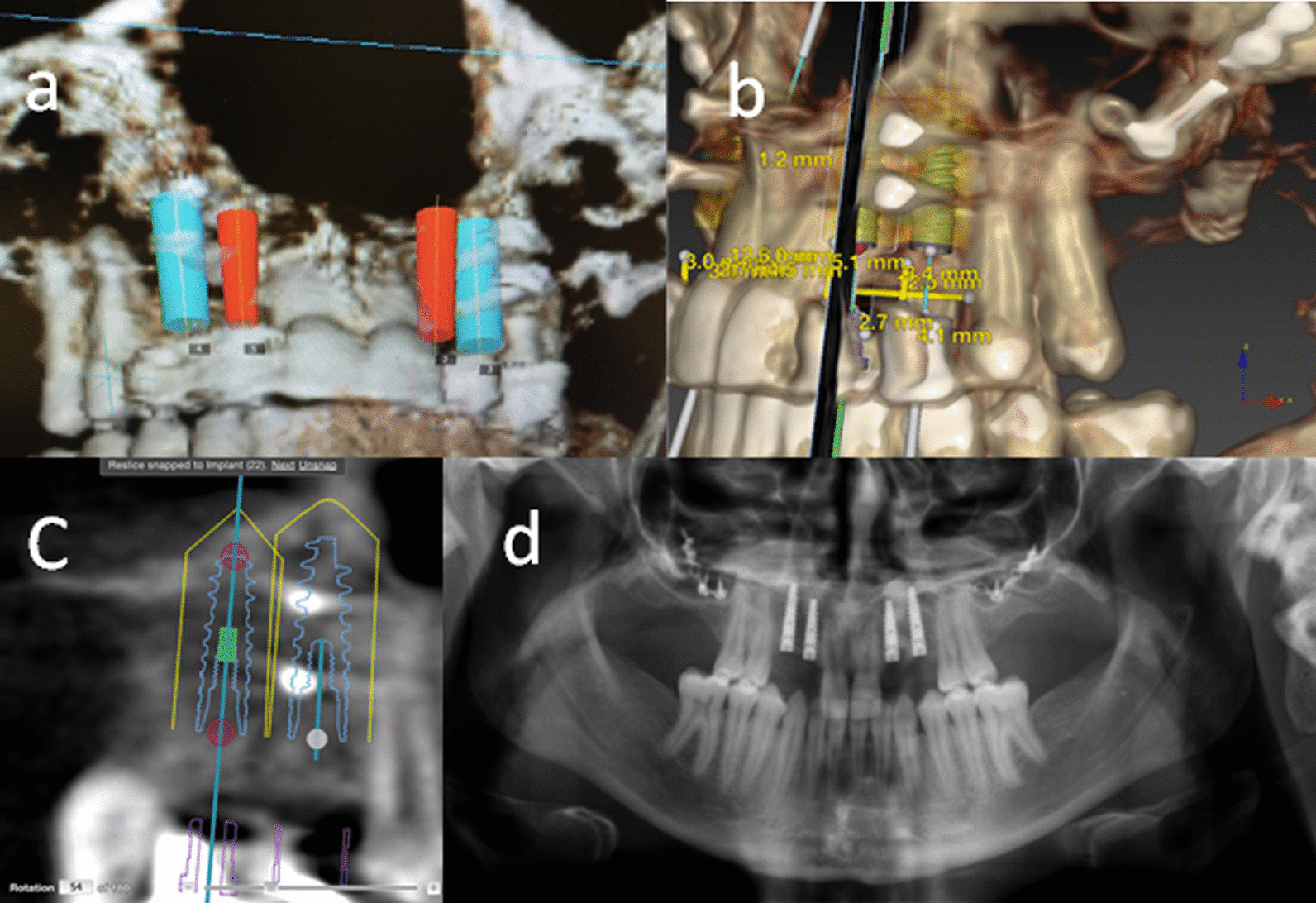
Implant placement: **a**–**c** Implant placement planning using CBCT with radiographic guide. **d** Panoramic radiograph showing implant installation

Four months later, a cone beam computer tomography scan was obtained and an implant planning software (NobelClinician, Nobel Biocare, USA) was used to determine implant position and size (Fig. 5a–c). Given the limited mesio-distal space, 3 mm diameter implants were chosen to preserve blood supply and ensure adequate space between implants and adjacent tooth roots. During surgery, lag screws placed previously were removed, 3.0 × 13 mm implants (Nobel Active) were placed at sites #6 and 11, and 3.0 × 11.5 mm implants (Nobel Active) were placed at sites #7 and 10 (Fig. 5d). Sub-crestal implant placement was achieved with 30 Ncm of torque. Five months later, the stage 2 implant surgery was carried out to expose the implants, which were protected with healing abutments thereafter.

**Fig. 6:**
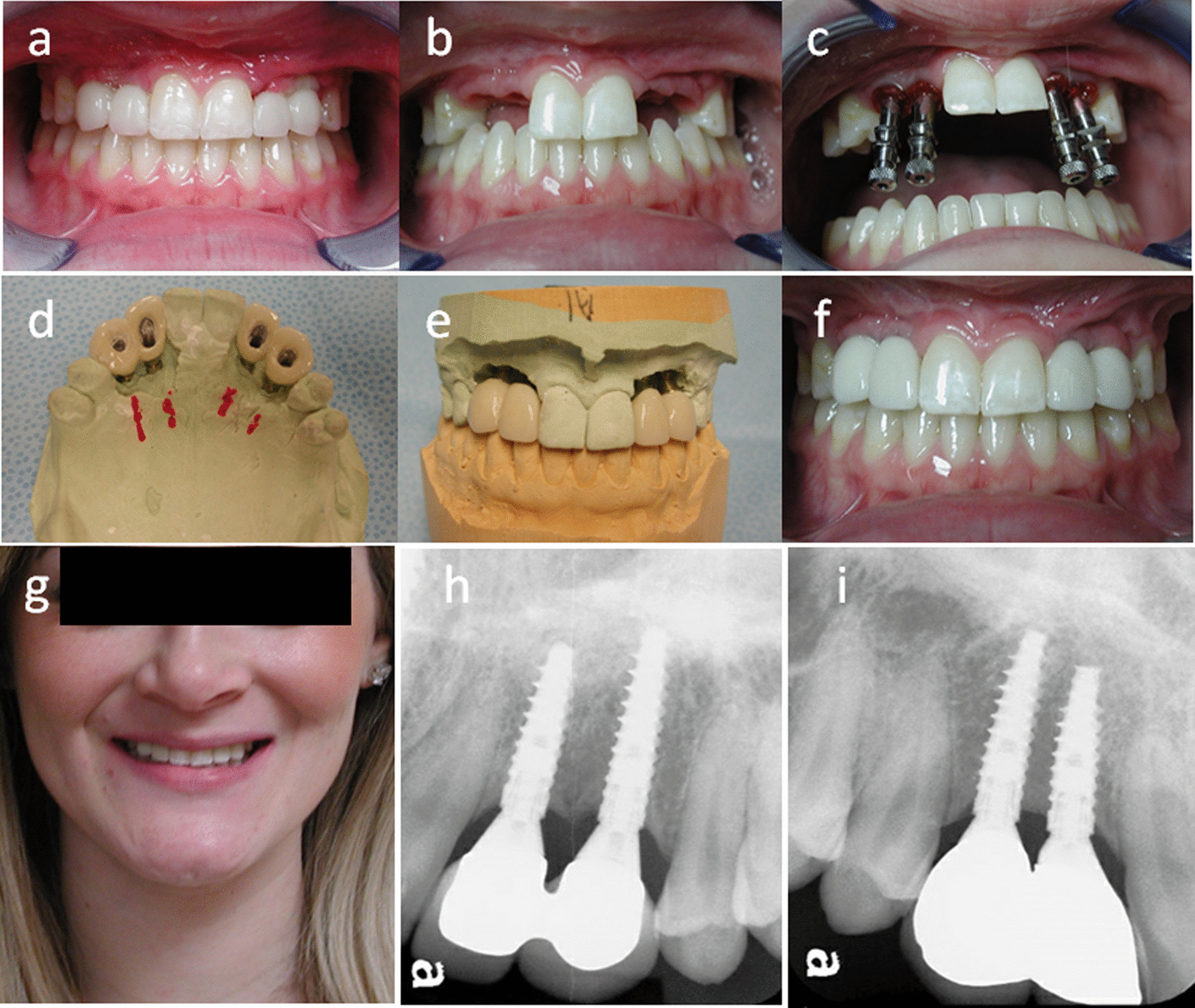
Prosthodontic rehabilitation: **a** Provisional prostheses: notice the gingival level was very low at initial stage. **b** Transmucosal tissue shaped by provisional prosthesis. **c** Implant level impression was taken with custom impression coping. **d** and **e** Screw-retained definitive prostheses on cast. **f** Intraoral view of final prostheses. **g** Extraoral view at the end of treatment. **h** and **i** Periapical radiographs in 3-year follow-up

The provisional implant crowns were used to facilitate the establishment of desirable emergence profile of the transmucosal tissue around the implant restorations (Fig. 6a). After 2 months, the transmucosal tissue matured to satisfactory extent (Fig. 6b) and the permanent implant prosthesis fabrication was thus initiated. Custom impression copings were used to register and transfer the shape of pre-formed transmucosal tissue to an implant level impression (Fig. 6c). Definitive prostheses were fabricated accordingly with custom abutments and ceramic crowns. The careful surgical planning and precision in execution resulted in the placement of implants at the desirable positions, which allowed us to fabricate screw-retained prostheses (Fig. 6d, e). To increase the strength and durability, the implant crowns of lateral incisor and canine were splinted on both sides. Patient was very satisfied with the esthetic outcome (Fig. 6f, g). Patient presented with continuous satisfaction with stable osseointegration in 3-year follow-up (Fig. 6 h, i).

## Discussion

In the treatment of patients with congenitally missing teeth, dental implants have become a widely accepted treatment option. However, our patient presented with oligodontia in association with additional significant oral maxillofacial conditions including maxillary hypoplasia, malar deficiency, malocclusion, and bone deficiency. Given the complexity of patient’s oral and maxillofacial condition, dental implant treatment alone cannot achieve an optimized esthetic and functional rehabilitation. Thus, a multidisciplinary and staged approach is warranted.

The patient presented in this case report initiated her clinical treatment at the age of 14-year-old. The majority of females reach skeletal maturity at 16 years of age. Thus, we determined that the reverse-pull facemask therapy, which is a standard protocol in the early management to assist the growth of maxilla and correct retrognathic maxilla for children [20, 21], would not be beneficial to her since she was approaching skeletal maturity. Before treatment, our patient had a skeletal Class III malocclusion with labially flare maxillary anterior teeth, a common compensation in maxillary deficiency patients. After the evaluation by the orthodontist and oral maxillofacial surgeon, pre-operative orthodontic decompensation therapy was performed to align anterior teeth in ideal position relative to the alveolar base. After the completion of pre-surgical orthodontic treatment, a high Le Fort I maxillary osteotomy was successfully performed to advance and downgraft to ideally position the maxilla with respect to the alveolar base.

In the literature, sinus augmentation has been described to be performed simultaneously with Le Fort I maxillary osteotomy in cases where maxillary posterior implants are desirable as part of the overall restorative plan [18, 22]. However, the latter combined approach may carry a higher risk of infection. In this case, we did not perform sinus augmentation to place posterior implants. Instead, a premolar occlusion was provided to the patient, aiming to simplify the treatment and reduce the cost and risk. Importantly, during the long-term provisional restoration phase with both removable partial denture and interim implant prostheses, the patient reported a satisfactory masticatory function with premolar occlusion. At the 4 years follow-up, the patient continued to report that she had been functioning well with provided premolar occlusion, which is consistent with the literature documenting the success of shortened dental arch option [23, 24].

Due to lack of teeth eruption, alveolar bone is typically underdeveloped in patients with oligodontia. Insufficient bone quantity brings enormous challenges and risks to implant therapy such as inadequate initial stability and peri-implant bone loss in the long term [25]. Therefore, the need of bone augmentation in oligodontia patients’ implant therapy is more often than general population. One report showed that more than 70% oligodontia cases require alveolar ridge augmentations [19]. For our patient, a ridge preservation graft and a second ridge augmentation graft were required. The former was performed at the time that primary teeth C, D, G, H were extracted with particulate bone graft, and the latter was carried out with allograft block bone grafts. Of note, in the treatment of our patient, we used Infuse™ bone graft, which contains human bone morphogenetic protein-2 (rhBMP-2). BMP-2 is a potent bone inducer, originally identified through its ectopic bone formation abilities [26]. It was approved by FDA as an alternative to autogenous bone grafts for sinus augmentations, and for localized alveolar ridge augmentations [27]. The bone graft treatment for our patient achieved desirable outcome and led to the subsequent successful implant placement.

**Fig. 7:**
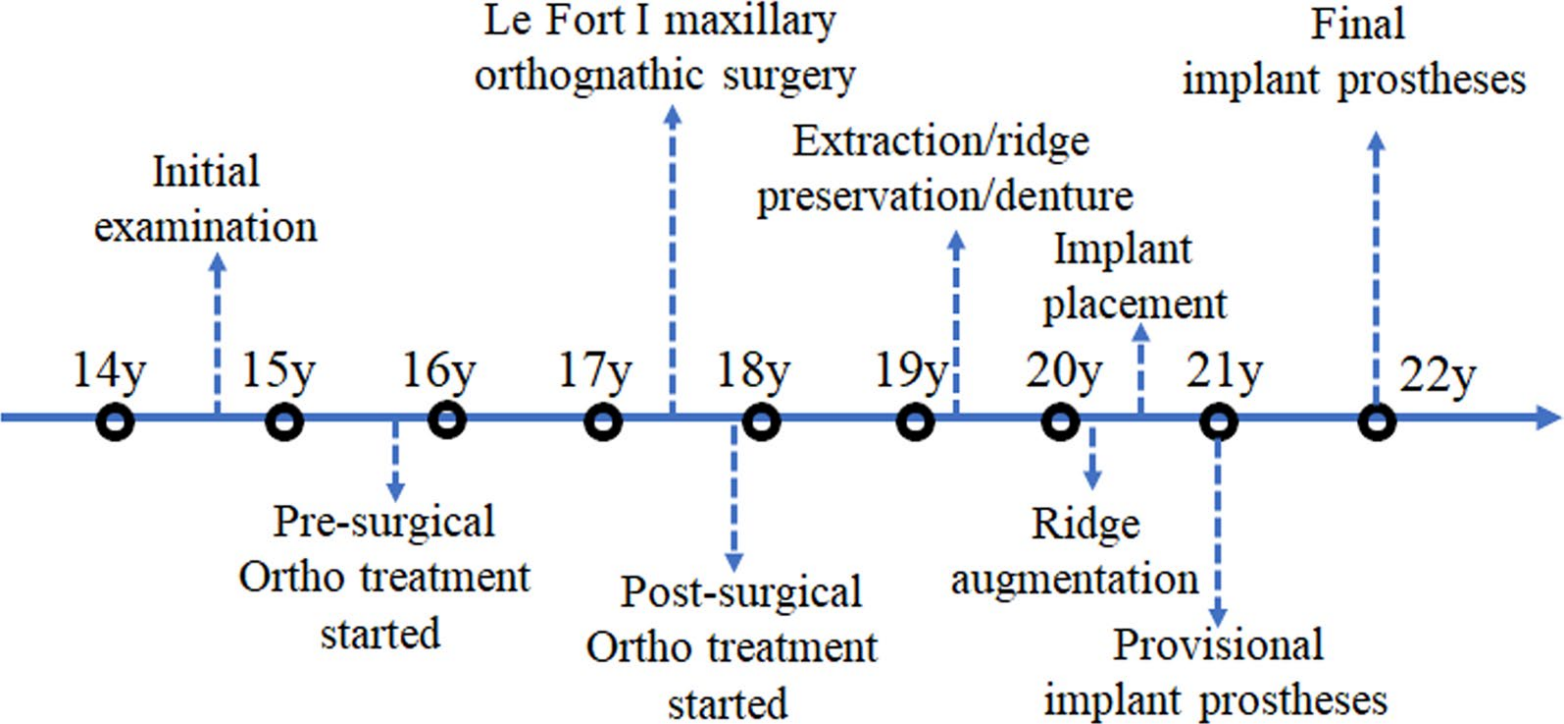
Treatment timeline

In this case report, we described the successful dental management of an oligodontia patient with complex dentofacial abnormalities. Collaborative interdisciplinary care by orthodontist, maxillofacial surgeon, and prosthodontist was required to successfully restore oral function, form, and comfort and achieve desirable outcome. Given the long treatment duration, clinicians must be mindful to avoid patient burnout by actively involving the patient and parents in open discussion about treatment options and goals at different stages of treatment. For this patient, it took 7 years to finish the treatment (Fig. 7). The patient and her parents had been actively engaged in each phase of treatment. The goal and result of each phase were clearly communicated and reviewed with patient and her parents. Patient understood the predicted result of each step and appreciate the importance of each procedure. At the end of each treatment phase, the result and the plan of next phase were reviewed with patient and her parents so they appreciated the progress and understood what to expect next, thus, patient was motivated to continue the treatment. To optimize the prosthetic result, we used diagnostic teeth set-up, interim removable partial denture, and interim implant prostheses to streamline the treatment at the different treatment phases. The ability to continuously provide patient satisfactory temporary prostheses played a key role in the successful execution of this long-term treatment.

## Conclusions

The successful treatment of oligodontia patient with complex dentofacial abnormalities requires the close and orderly collaboration among orthodontist, oral maxillofacial surgeon, and prosthodontist. Within the limitations of this case report, presented interdisciplinary approaches may optimize the oral rehabilitation outcome in patients with similar clinical challenges. A prospective clinical investigation is desired to verify the benefit of presented interdisciplinary approach.

## Data Availability

Not applicable.

## Abbreviations

rhBMP-2: Recombinant human bone morphogenetic protein-2
